# How Executive Processes Explain the Overlap between Working Memory Capacity and Fluid Intelligence: A Test of Process Overlap Theory

**DOI:** 10.3390/jintelligence9020021

**Published:** 2021-04-06

**Authors:** Tengfei Wang, Chenyu Li, Xuezhu Ren, Karl Schweizer

**Affiliations:** 1 Department of Psychology and Behavioral Sciences, Zhejiang University, Hangzhou 310028, China; lichenyu6020@outlook.com; 2 School of Education, Huazhong University of Science and Technology, Wuhan 430074, China; renxz@hust.edu.cn; 3 Department of Psychology, Goethe University Frankfurt, 60323 Frankfurt, Germany; K.Schweizer@psych.uni-frankfurt.de

**Keywords:** working memory capacity, fluid intelligence, executive processes, fixed-links modeling

## Abstract

Working memory capacity (WMC) and fluid intelligence (Gf) are highly correlated, but what accounts for this relationship remains elusive. Process-overlap theory (POT) proposes that the positive manifold is mainly caused by the overlap of domain-general executive processes which are involved in a battery of mental tests. Thus, executive processes are proposed to explain the relationship between WMC and Gf. The current study aims to (1) achieve a relatively purified representation of the core executive processes including shifting and inhibition by a novel approach combining experimental manipulations and fixed-links modeling, and (2) to explore whether these executive processes account for the overlap between WMC and Gf. To these ends, we reanalyzed data of 215 university students who completed measures of WMC, Gf, and executive processes. Results showed that the model with a common factor, as well as shifting and inhibition factors, provided the best fit to the data of the executive function (EF) task. These components explained around 88% of the variance shared by WMC and Gf. However, it was the common EF factor, rather than inhibition and shifting, that played a major part in explaining the common variance. These results do not support POT as underlying the relationship between WMC and Gf.

## 1. Introduction

Positive manifold is one of the most replicated findings in intelligence research (Carroll 1993). It describes a pattern of positive correlations among different cognitive tests (Spearmen 1904). A number of theories have been proposed to explain such a pattern. For instance, Spearmen (1904) proposed that different cognitive tests correlate because they all measure a single latent factor. In contrast, Thomson’s (1916) sampling theory considers that the correlation between any two cognitive tests is simply the function of the number of “bonds” the tests share. Bonds could be either higher-order general processes which play a role in different cognitive activities or lower-order specific processes which are only involved in specific tasks. However, a recently proposed theory of intelligence, namely, process overlap theory (POT), claims that positive manifold is mainly due to domain-general processes, which act as a bottleneck constraining performance in a variety of cognitive tasks (Kovacs and Conway 2016). Furthermore, they speculate that those domain-general processes are a limited number of elementary executive processes, which affect performance on many cognitive tests. Following POT, the positive relationships between cognitive ability tasks, including Gf and WMC tasks, are mainly due to individual differences in executive processes such as updating, inhibition, or shifting (Miyake et al. 2000).

Working memory refers to a capacity-limited system enabling the simultaneous maintenance and manipulation of information (Baddeley 1992). The capacity of working memory has been shown to predict performance for a number of higher-order cognitive abilities, such as intelligence (Kyllonen and Christal 1990), complex learning (Wang et al. 2013, 2015), and reading comprehension (Daneman and Carpenter 1980). Among these abilities, WMC’s predictive power of Gf has received a considerable amount of attention. Gf refers to the ability to solve novel and complex problems by means of mental operations such as identifying relationships, drawing inferences, and so forth, which are relatively less influenced by educational and cultural factors (Cattell 1963). Meta-analytic studies have demonstrated that WMC and Gf share around 50% to 85% of their latent variance (Kane et al. 2005; Oberauer et al. 2005). However, the mechanism underlying the overlap between WMC and Gf remains elusive. A line of previous studies focused on the role of inhibition (or attention control) in the relationship between WMC and Gf (see Chuderski et al. 2012; Shipstead et al. 2014; Unsworth et al. 2014), but the nature of the involvement of different executive processes in WMC and Gf has not been thoroughly specified. The present study, therefore, attempts to assess major executive processes by means of a novel approach combining experimental manipulations and statistical modeling, and it further investigates the extent to which those processes contribute to the overlap between WMC and Gf.

Executive processes derive from the concept of executive function (EF), which has been conceptualized as a supervisory system that is responsible for the coordination and control of goal-directed behavior (Miyake et al. 2000). Due to its significance for everyday lives, EF has attracted a great deal of attention in many subdisciplines of psychological science (Miyake and Friedman 2012). In a seminal study examining the structure of EF, Miyake et al. (2000) identified three major EFs: updating, shifting, and inhibition, which are moderately related to each other, but are clearly separable. These processes serve different functions for goal-directed behavior. Updating is the process of replacing outdated and no-longer-relevant information with new and relevant information. This process enables the active manipulation of the contents in working memory, going beyond a simple storage of information. It also precludes working memory from being overloaded in considering its capacity limit (Morris and Jones 1990). Shifting refers to the process of flexibly switching between multiple operations, tasks, or mental sets (Monsell 1996). It requires not only the ability to engage or disengage appropriate task sets, but also the capacity to execute a new operation in the face of proactive interference elicited by previous task sets. Inhibition has also been referred to as attention control (Engle and Kane 2004; Unsworth et al. 2014). It involves the process of deliberately suppressing automatic or prepotent responses, and resisting external or internal interferences that may distract one’s attention away from the ongoing task (Friedman and Miyake 2004).

All three executive processes are assumed to be important for WMC. As depicted in the working memory model proposed by Baddeley and Hitch (1974), there is a domain-general “central executive” responsible for the coordination of multiple tasks. It shares similar features to EF, such as selectively attending to relevant information while avoiding interferences, flexibly switching between the processing of secondary tasks and attentional refreshment of memory traces, and the active updating of information. However, empirical studies have revealed that not all EFs are comparably related to WMC and Gf. Miyake et al. (2000) examined the relationship between three EFs and WMC measured by operation span. They found WMC to be highly associated with updating, but not with shifting or inhibition. This result was confirmed by another study indicating a high latent correlation (*r* = .96) between updating assessed by *n*-back tasks and WMC tapped by a set of content-heterogeneous tasks (Schmiedek et al. 2009). In contrast, there are also experimental and correlational studies suggesting a relatively weak relationship between WMC and updating (see Redick and Lindsey (2013) for a meta-analysis). For example, practice on the *n*-back task does not improve performance on the other WMC measures (e.g., Jaeggi et al. 2008; Redick et al. 2013). Additionally, Kane et al. (2007) reported nonsignificant to weak correlations between WMC and updating tasks.

Recently, there have been considerable debates as to whether inhibition (or attention control) is related to WMC (e.g., Draheim et al. 2020; Rey-Mermet et al. 2019; Unsworth et al. 2020; Wang et al. 2020). On one hand, a large body of studies suggest a moderate to high correlation between inhibition and WMC (Draheim et al. 2020; Unsworth et al. 2020). In a recent study, Unsworth et al. (2020) pooled data from multiple studies, whereby their analyses on the combined dataset suggested that the latent inhibition factor is consistently associated with WMC. On the other hand, there were also studies even casting doubt on the existence of inhibition as a psychometric construct since inhibition tasks show only weak and near-zero correlations. Furthermore, these individual tasks of inhibition were unrelated to WMC and Gf (Rey-Mermet et al. 2018, 2019). Although it is theoretically sound to expect a relationship between shifting (or task switching) and WMC, numerous studies have failed to reveal this relationship (Draheim et al. 2016; Miyake et al. 2000; Oberauer et al. 2003). For example, Oberauer et al. (2003) reported correlations ranging from −.07 to .23 between task switching and six WMC tasks. However, Draheim et al. (2016) found that strong relationships are obtained when a new scoring method that integrates reaction time and accuracy into a single score is used as the dependent variable of shifting.

According to the theoretical account of Gf, solving problems in typical Gf tests (e.g., Raven’s matrices) requires the identification and subsequent application of abstract rules (Carpenter et al. 1990). During such complex mental processing, the ability to inhibit irrelevant or competing information from entering into working memory is an important precondition for arriving at correct solutions since irrelevant features, rules, or response alternatives may divert one’s attention to an incorrect answer (Jarosz and Wiley 2012). Meanwhile, when one rule is proven to be incorrect, one has to flexibly switch attention toward a new one. Friedman et al. (2006) investigated to what extent updating, shifting, and inhibition predicted Gf by means of a latent variable approach. Results showed that only updating predicted Gf (*β* = .74). The path coefficients from inhibition (*β* = −.11) and shifting (*β* = −.08) to Gf were not significant. On the other hand, recent studies suggested that there were significant correlations between shifting (Wang et al. 2013) and inhibition (Wang et al. 2020) with Gf when EFs were represented by a different approach.

Since executive processes play important roles in both WMC and Gf, it seems reasonable to assume that executive processes may underlie the overlap between WMC and Gf. There are already studies exploring to what extent inhibition contributes to the relationship between WMC and Gf (Chuderski et al. 2012; Unsworth et al. 2014). Chuderski et al. (2012) found that individual differences in attention control, interference resolution, and response inhibition fail to account for the Gf–WMC link. Unsworth et al. (2014) suggested that attention control partly accounts for the relationship between WMC and Gf, in addition to storage capacity and secondary memory retrieval. Although these studies shed light on the role of inhibition in explaining the mechanism underlying the WMC–Gf overlap, it remains unclear the extent to which inhibition, along with updating and shifting, accounts for the overlap. According to POT, it is expected that executive processes would explain unique portions of the overlap between WMC and Gf.

To sum up, the current study aims to test POT by examining how and to what extent domain-general executive processes account for the relationship between WMC and Gf. The structural investigation by Miyake et al. (2000) provides a theoretical framework for organizing executive processes, with a focus on updating, shifting, and inhibition. However, the conventional measures of EFs are largely vexed by the task-impurity problem. That is, the task not only taps the executive process of interest, but also the other non-EF processes. The task impurity may lead to distortions in the investigation of the relationship between specific cognitive processes or constructs (Schweizer 2007). To solve the task-impurity problem, we developed a single experimental paradigm in which two executive processes, namely, shifting and inhibition were manipulated simultaneously. The systematic changes of variances in performance led by specific manipulations can be captured by fixed-links modeling (Schweizer 2008).

## 2. Methods

### 2.1. Participants

The same sample provided data for investigating another research question, which focused on individual differences in Gf (Wang et al. 2017). A total of 228 participants were recruited from a university in central China. Thirteen participants were excluded for not completing the star counting task (SCT). This left a final sample of 215 participants (101 males and 114 females) aged between 18 and 24 years (*M* = 20.93, *SD* = 1.14). All participants received a financial reward for participation.

### 2.2. Measures

#### 2.2.1. Executive function task

**Figure 1:**
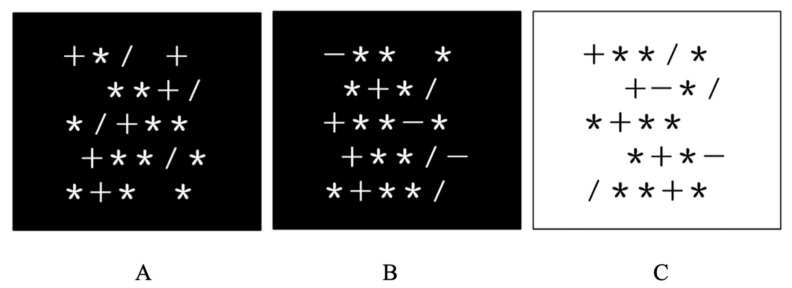
Illustration of the star counting task: (**A**) screen used in the first treatment level with only plus signs; (**B**) screen used in the second treatment level with both plus and minus signs; (**C**) screen used in the third treatment level with white background indicating the reversal of the meaning of plus and minus signs.

The original star counting task (SCT, de Jong and Das-Smaal 1995; Ren et al. 2013; Wang et al. 2015) was modified to tap executive processes. The task asked participants to mentally count the number of stars in a forward or backward manner starting from a given number. The counting stimulus was a 14 × 16 cm rectangle including a few stars interleaved with plus, minus, and slash signs (see Figure 1). The direction of counting was determined by the plus or minus signs. The slashes were meaningless and were to be neglected. There were five rows of symbols in each rectangle. Each row consisted of three to five symbols which were not vertically aligned.

In each trial, participants were instructed to press the “enter” key after preparing themselves for taking the item. Then, the starting number (from the range between 12 and 30) appeared on the screen for 1 s, followed by the described rectangle with symbols. Participants were to count the stars as quickly and as accurately as possible row by row. The rectangle remained onscreen until the participant pressed the “enter” key but no longer than 40 s. Afterward, the participants were asked to enter the final number into a box presented in the center of the screen.

Three treatment levels were designed to stimulate different types of executive processes. The first level included plus signs only, and the second and third levels included both plus and minus signs. Compared to the first level, the other two levels additionally demanded shifting since participants had to switch between forward and backward counting. In addition, the third level differed from the second level in the counting rule which required participants to count the number of stars in a backward manner after coming across a plus sign but in the forward manner after seeing a minus sign. The change of the rule was indicated by the change in colors of symbols and background. White symbols together with a black background signified the outset state of the rule (see Figure 1A,B). The reversal of the counting rule was signified by black symbols together with a white background (See Figure 1C). The reversal of the overlearned rule was supposed to place extra demands on inhibition. All trials required participants to track and count the relevant stimuli (i.e., stars) while updating the number of stars in working memory. There were five practice trials and 36 experimental trials. The sequence of trials from different levels was pseudo-randomized. The dependent variable was the percentage of trials completed correctly in each level.

#### 2.2.2. Working Memory Capacity Tasks

The complex span task (CST) and the Brown–Peterson task (BPT) were used to tap WMC (Wang et al. 2017). The participants’ task was to remember a series of letters while performing a set of computer-paced secondary tasks. The letters were 19 consonants (except for L and W). The secondary task was to decide whether a word was an animal noun or not. CST and BPT differed in the placement of letters and secondary tasks. To be specific, the secondary tasks were interleaved with the letters in CST. In contrast, the secondary tasks followed the presentation of all the to-be-remembered letters in the BPT.

Each trial started with the presentation of a fixation point for 750 ms. This was followed by 250 ms of a blank screen. In CST, each letter was presented for 750 ms, followed by a blank screen for 250 ms and a period during which either two or four words were presented within 3700 ms. Lastly, participants had to recall the letters in the same order as they were presented. The number of letters varied randomly among three, five, and seven. There were eight trials of each set size.

In BPT, letters were presented in succession. Each letter was shown for 750 ms with a 250 ms inter-stimulus interval (ISI). After all the letters were displayed, an asterisk appeared for 250 ms, followed by a set of secondary tasks, during which four or eight words were presented for 7400 ms. Then, the letters were to be recalled in the same order as they were presented. There were three, five, or seven letters in each trial. Each list length was applied in 12 trials. The dependent variable was the percentage of correct letters recalled in the correct position.

#### 2.2.3. Fluid Intelligence Measures

We used Cattell’s culture fair test (CFT; Cattell 1971) and Horn’s abstract reasoning test (ART; Horn 1983) to assess fluid intelligence. CFT comprised four subtests, namely, series, classifications, matrices, and topologies. Participants were allowed to complete each subtest within 2.5–4 min. The total number of correctly solved items across the four subtests was calculated. ART comprised 40 items presented in ascending order of difficulty. In each item, there was a series of nine numbers or letters, in which eight followed a rule but one did not. The participants’ task was to infer the rule and identify the inappropriate number or letter. The time limit was set to 8 min. The total number of items solved correctly was used as the dependent variable.

### 2.3. Procedure

Participants first completed CFT and ART, followed by BPT, SCT, and CST. The measures of Gf were paper-and-pencil tests, while the tasks of WMC and executive processes were computerized and presented on a 19 inch monitor with E-prime. It took approximately 1 h to complete these measures. Participants were allowed to have a short break between tasks.

### 2.4. Modeling Analysis

We used LISREL 8.8 (Jöreskog and Sörbom 2006) for the statistical investigation of models. Parameters were estimated using the maximum-likelihood estimation method. The fit statistics were evaluated on the basis of criteria recommended by Kline (2015) and DiStefano (2016). Specifically, the model fit was considered good (or acceptable) if normed *χ*^2^ (= *χ*^2^/df) ≤ 2 (3), root mean square error of approximation (RMSEA) ≤ .06 (.08), standardized root mean square residual (SRMR) ≤ .08 (.10), and comparative fit index (CFI) ≥ .95 (.90). Furthermore, models were compared according to Akaike’s information criterion (AIC), with a smaller AIC indicating a better model fit. The models are described in detail together with the results.

## 3. Results

### 3.1. Descriptive Statistics

Table 1 presents the descriptive statistics of three treatment levels of SCT, the two WMC tasks, the two fluid intelligence tests, and the intercorrelations between them. The mean accuracy scores of the three treatment levels of SCT decreased from the first to the third levels. A repeated-measure analysis of variance indicated significant differences between the three treatment levels, *F* (2, 428) = 49.48, *p* < .001, partial *η*^2^ = .19. Post hoc tests revealed that the mean accuracy of the first level was significantly higher than the second and third levels (*p*s < .001), while the second and third levels did not differ in the scores (*p* = .30).

### 3.2. The Relationship between WMC and Gf

**Figure 2:**
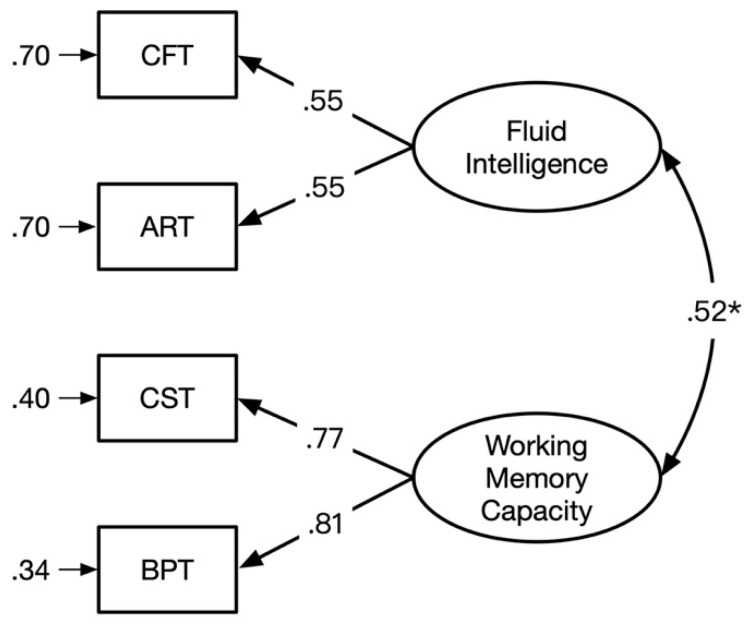
The two-factor model including fluid intelligence and working memory capacity as two distinct but correlated constructs. The culture fair test (CFT) and abstract reasoning test (ART) were loaded on fluid intelligence, and the complex span task (CST) and Brown–Peterson task (BPT) were loaded on working memory capacity (* *p* < .05).

To examine whether WMC and Gf are isomorphic (Martínez et al. 2011) or not (Ackerman et al. 2005), a one-factor model and a two-factor model were established. In the one-factor model, all measures of WMC and Gf were loaded on a general factor. In contrast, the WMC factor was derived from scores of CST and BPT and the Gf factor was derived from scores of CFT and ART in the two-factor model (see Figure 2). As seen from the model fit statistics in Table 2, the one-factor model was not acceptable, while the two-factor model showed good fit. The latent correlation between WMC and Gf was *r* = .52, indicating that the two constructs shared more than 27% of their latent variances. Although the Gf–WMC correlation was substantial, it was not large enough to warrant unity between WMC and Gf, as shown by the model fit statistics.

### 3.3. Representation of the Executive Processes

**Figure 3:**
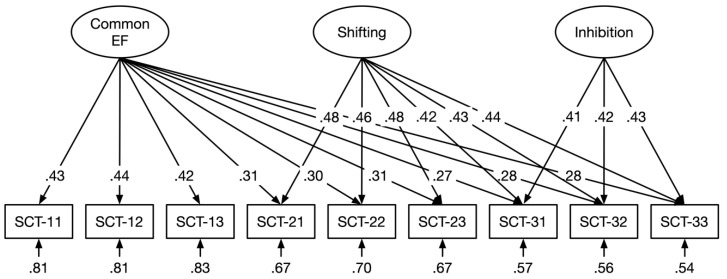
The three-factor model of the star counting task (SCT) with three latent variables representing common EF, shifting, and inhibition.

In order to represent the executive processes by fixed-links modeling, three sub-scores for each treatment level of SCT were generated by combining scores of four trials (see Wang et al. 2015). We constructed a standard CFA model and three fixed-links models according to the assumed executive processes stimulated by the experimental manipulations. Model 1 was a conventional CFA model including a single factor derived from all scores, with the factor loadings being freely estimated. Models 2–4 were fixed-links models, in which the loadings on each latent variable were fixed to one, while the variances of latent variables were freely estimated (Ren et al. 2013). The correlations between latent variables were constrained to zero (Schweizer 2008). Specifically, model 2 included a single factor derived from all scores, and all factor loadings were fixed to one. Model 1 and model 2 assumed that a common factor was sufficient to explain the variations in scores across all treatment levels. Model 3 comprised a common EF factor and a shifting factor. The scores of the second and third treatment levels were loaded on the shifting factor. Model 4 included common EF, shifting, and inhibition factors, with the scores of the third treatment level loaded on the inhibition factor (see Figure 3). The common EF factor might partly reflect updating, i.e., the process of monitoring and renewing the number of stars in working memory. However, it was not prevented that other processes contributed to performance on this task. In contrast, the shifting and inhibition factors indicated the cognitive processes specific to each construct. Specifically, shifting reflected the process of flexibly switching between plus and minus operations, and inhibition reflected the process of resisting prepotent responses to the plus and minus signs. Table 2 shows the fit statistics of the four models. It can be seen that only the three-factor model had a good model fit, whereas the others did not. The scaled variances of all three latent variables were significant (common EF: *φ* = .047, *t* = 4.77, *p* < .001; shifting: *φ* = .074, *t* = 5.21, *p* < .001; inhibition: *φ* = .036, *t* = 3.74, *p* < .001), confirming the presence of three components underlying SCT.

### 3.4. Explaining the Relationship between WMC and Gf with Executive Processes

**Figure 4:**
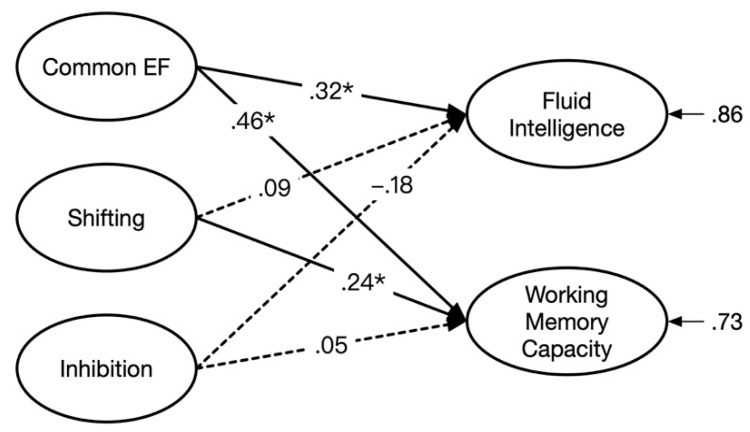
The prediction of fluid intelligence and working memory capacity with three latent factors underlying the star counting task including common EF, shifting, and inhibition as predictors (* *p* < .05).

We established a comprehensive model including executive processes according to Model 4 as predictors of WMC and Gf. This comprehensive model showed a good fit to the data, *χ*^2^ (66) = 103.34, *p* = .002, *χ*^2^/*df* = 1.57, RMSEA = .051, SRMR = .075, and CFI = .95. Figure 4 presents the illustration concerning the prediction of WMC and Gf by the three SCT-based latent variables. The inspection of the standardized regression weights suggested that common EF had a substantial effect on both WMC (*β* = .32, *t* = 2.99, *p* < .01) and Gf (*β* = .46, *t* = 4.43, *p* < .01). Shifting was significantly predictive of WMC (*β* = .24, *t* = 2.39, *p* < .05). However, the other regression weights were not significant.

Lastly, in order to obtain a more direct representation of the overlap (between WMC and Gf) that was explained by executive processes, we employed a statistical approach for investigating how much variance common to both criterion variables was left unexplained by the predictors (see Chuderski et al. 2012; Colom et al. 2008). Specifically, according to the model presented in Figure 4, the correlation between the residual variances of WMC and Gf was freely estimated. The model fit of this modified model was good, *χ*^2^ (65) = 99.65, *p* = .004, *χ*^2^/*df* = 1.53, RMSEA = .050, SRMR = .070, and CFI = .95. The residual correlation was merely *r* = .18, *t* = 1.96, *p* = .054, suggesting that WMC and Gf no longer overlapped after the variance due to executive processes was accounted for. In considering the original latent correlation between WMC and Gf (*r* = .52), this result suggests that executive processes accounted for a large part of the variance (i.e., .88 = 1 − .18^2^/0.52^2^) shared by WMC and Gf. Moreover, the common EF factor mainly explained the overlap.

## 4. Discussion

A recent theory of intelligence, namely POT, claims that the positive manifold is mainly due to the extent to which cognitive tests tap domain-general executive processes. This theory serves as the starting point of the current study that focused on two highly correlated cognitive constructs, i.e., Gf and WMC, and examined to what extent their overlap was accounted for by executive processes. First, in order to measure the executive processes, the star counting task was modified to include three treatment levels with varying demands on two executive processes (Ren et al. 2013; Wang et al. 2013). Fixed-links models were used to decompose the variances of performance on the task into three components: common EF, shifting, and inhibition. Results showed that the model including three latent factors provided the best model fit. Second, these factors were linked to WMC and Gf. Results showed that common EF and shifting contributed to WMC, while Gf was only predicted by common EF. A further analysis indicated that common EF accounted for most of the shared variance between Gf and WMC.

The comparison of models on the relationship between Gf and WMC suggests that Gf and WMC are related but separate cognitive constructs. WMC showed medium-sized correlation (*r* = .52) with Gf. This result echoes the conclusion that WMC and Gf are not isomorphic constructs (Ackerman et al. 2005; Kane et al. 2005), whereas it is inconsistent with Kyllonen and Christal (1990) and Martínez et al. (2011) suggesting that WMC or short-term memory is near-perfectly correlated with Gf. This result provides a precondition for further exploring the mechanism underlying their overlap.

As for the measurement of executive processes, we adopted a relatively new approach, assessing core components of EF within a single framework. The main advantage of this approach is that executive processes can be statistically represented according to the experimental manipulation. That is, by contrasting the treatment levels involving shifting or not, we could extract the shifting factor. By contrasting the treatment levels involving inhibition or not, the inhibition factor could be specified. The modeling results confirmed the validity of our manipulations, showing that the three-factor model provided better model fits than the others. Our model is more in line with Friedman and colleagues’ (2008, 2017) bifactor model including a common EF factor and two EF-specific factors, but it differs from their previous model including three correlated EF factors (Miyake et al. 2000). Although both models highlight the nature of unity and diversity in EF, unity and diversity are represented differently in these models. In the correlated-factor model, unity and diversity are reflected by the magnitudes of the correlations between different EF factors. Factor correlations larger than zero would indicate that there is some common ground, and correlations smaller than one would indicate some diversity despite a high degree of communality. On the other hand, in the bifactor model, unity is captured by the common EF factor that is derived from all tasks, and diversity is captured by the shifting-specific and updating-specific factors that are extracted from the remaining shifting and updating tasks, respectively. Once the correlations due to the common EF factor are isolated, the shifting and updating factors are orthogonal to the common factor and each other (Friedman and Miyake 2017). However, it should be noted that the common EF factor in our study differs from that in Friedman et al. (2008). In their studies, there was no inhibition-specific factor since the common EF factor accounted for all the correlations among the inhibiting tasks. In our study, the common EF factor was extracted from all three treatment levels, and two nested factors were extracted from treatment levels assessing shifting and inhibition, respectively. Although there was no updating factor, the processes of tracking and updating the number of stars in working memory are fundamental to completing trials across all treatment levels. Therefore, we contend that updating might be embedded in the common EF factor. This theoretical assumption was validated in another study, in which the common factor derived from two treatment levels in the star counting task was associated with an established updating task, the exchange test (Ren et al. 2013). Their result showed that the common factor was substantially related to the exchange test scores (*r* = .53). In addition, the shifting and inhibition factors reflect the cognitive processes specific to each construct. Specifically, shifting may reflect the process of switching flexibly between different task-set representations (i.e., from plus to minus or the reversal), and inhibition may reflect the process of resisting prepotent responses to the plus and minus signs (Friedman and Miyake 2004).

A close inspection of the link between executive processes with Gf and WMC indicates that only the common EF factor plays a role in both constructs, while shifting and inhibition do not. These results are consistent with the previous findings of Miyake et al. (2000) and Friedman et al. (2006), suggesting that only the ability to maintain task-relevant information, to remove the information when it becomes irrelevant, and to replace it with new information are essential in completing both WMC and Gf tasks. In a later study, Friedman and Miyake (2017) reported that the common EF (.51), updating-specific (.49), and shifting-specific (−.24) factors all had significant correlations with g, which was operationalized as the Wechsler Adult Intelligence Scale-third edition (WAIS-III) full-scale IQ. This result also suggests that updating and related EF processes are essential to intelligence. However, it should be noted that we only focused on Gf instead of g. Although shifting showed a significant relationship with WMC, it was unrelated to Gf. In completing WMC tasks, one has to frequently switch attention between the processing of secondary tasks and the rehearsal of to-be-memorized items (Barrouillet et al. 2004). Therefore, more efficient switches lead to a greater likelihood of refreshing the items and memorizing more information. On the other hand, such a process seems nonessential in completing a reasoning task. This result might be due to the fact that WMC tasks were more time-constrained than Gf tasks in our study. There is a relatively low demand with respect to the efficiency of shifting in the Gf tasks. Furthermore, inhibition was uncorrelated with either WMC or Gf. This result is inconsistent with previous studies (Draheim et al. 2020; Unsworth et al. 2020) probably because inhibition was represented differently in their studies, and its correlation with WMC or Gf might be due to the updating process, which is inevitably involved in performing a typical inhibition task. However, in our study the variances due to updating, shifting, and other general processes were separated from inhibition in the fixed-links model.

A novel finding of this study is that executive processes almost exhaust the common variance shared by Gf and WMC. However, it was mainly the common EF factor, rather than shifting and inhibition, that accounted for the shared variance. This result provides little support for POT. According to POT, the size of the correlation is a function of the overlap of multiple domain-general executive processes. In other words, if different executive processes really explained unique portions of the overlap between WMC and Gf, this result would support POT. Otherwise, if different executive processes did not account for the WMC–Gf covariation when their common variances are removed, this result would contradict the assumption of POT. In our study, it was exactly the latter case; only the common EF factor predicted both WMC and Gf, while the shifting and inhibition factors did not. Therefore, our results are not favorable of POT in this regard.

The current study may shed light on the EF training studies. Great efforts have been dedicated to examining whether short-term intensive training on specific EFs promotes intelligence and WMC. However, recent meta-analyses suggest that there is no convincing evidence of far-transfer effects of EF training (Kassai et al. 2019; Nguyen et al. 2019). In considering our finding that only the common EF contributed to higher-order cognitive abilities while the shifting-specific and inhibition-specific processes did not, EF trainings might be more effective if they target EF skills in general.

Meanwhile, the current study had a few limitations that are important to consider in future research. One limitation is that updating was not manipulated in the star counting task. Therefore, it was not possible to achieve a purified representation of updating in our model. Although the common EF factor partly reflects updating, other processes such as alertness, motor processes, and numerical processing may also play a role. Therefore, a future study could vary the demands on updating in the star counting task such that updating can be represented unambiguously in fixed-links modeling. Second, executive processes were assessed by a single experimental task. The shifting and inhibition indicators in our model may partly reflect task-specific processes. The task-specific effects were not removed by the common factor. This may limit comparability to previous studies not only because of the different method but also because of the task specificity. While there are merits of the approach combining experimental manipulation and fixed-links modeling, the current findings should be validated in future studies by designing similar tasks to assess executive processes. Furthermore, it is possible that sheer storage capacity or other non-EF processes could also dissolve the relationship between WMC and Gf (Colom et al. 2006; Martínez et al. 2011). Therefore, further investigations are warranted to approve or disapprove POT.

## Figures and Tables

**Table 1:** Descriptive statistics for all measures and the intercorrelations between them (*N* = 215).

Measure	*M*	*SD*	1	2	3	4	5	6
1. SCT1	.89	.11	-					
2. SCT2	.80	.16	.18 **	-				
3. SCT3	.77	.20	.33 **	.48 **	-			
4. CST	.80	.12	.27 **	.31 **	.35 **	-		
5. BPT	.73	.10	.07	.15 *	.14 *	.63 **	-	
6. CFT	26.30	3.82	−.03	.10	.10	.19 **	.24 **	-
7. ART	31.01	3.35	.16 *	.15 *	.08	.21 **	.22 **	.30 **

*Note:* SCT*i* = the *i*-th treatment level of the star counting task; CST = the complex span task; BPT = the Brown–Peterson task; CFT = Cattell’s culture fair test; ART = abstract reasoning test (* *p* < .05, ** *p* < .01).

**Table 2:** Fit statistics of the measurement models for working memory capacity, fluid intelligence, and executive processes based on the star counting task. WMC, working memory capacity; Gf, fluid intelligence; EF, executive function; AIC, Akaike’s information criterion.

Measures	Type of Model	*χ^2^*	*df*	*p*	*χ*^2^/*df*	RMSEA	SRMR	CFI	AIC
WMC and Gf	one-factor	12.14	2	.002	6.07	.154	.066	.93	28.14
	two-factor	.46	1	.500	.46	.000	.008	1.00	18.46
Star counting task	standard CFA model	61.19	27	<.001	2.27	.077	.063	.92	97.19
	one-factor	198.24	35	<.001	5.66	.148	.160	.71	218.24
	common EF + shifting	80.11	34	<.001	2.36	.080	.097	.90	102.11
	common EF + shifting + inhibition	57.78	33	.005	1.75	.059	.079	.95	81.78

## Data Availability

Data is available from the corresponding author upon reasonable request.
